# Comment on “MicroRNA-208a Silencing Attenuates Doxorubicin Induced Myocyte Apoptosis and Cardiac Dysfunction”

**DOI:** 10.1155/2016/7939626

**Published:** 2016-02-07

**Authors:** Vagner Oliveira-Carvalho, Edimar Alcides Bocchi

**Affiliations:** Núcleo de Insuficiência Cardíaca do Instituto do Coração do Hospital das Clínicas da Faculdade de Medicina da USP (InCor HC-FMUSP), 05403-900 São Paulo, SP, Brazil

In the last two decades, one family of regulatory RNAs called microRNAs (miRNAs) has fundamentally transformed our understanding of how gene networks are regulated. Currently, the study of miRNAs represents one of the most exciting areas of the modern cardiology research.

Unlike the wide range of RNAs encoded by the human genome this RNA variety has been noted for its unique ability to modulate an enormous and complex regulatory network of gene expression. To date, more than 2500 miRNAs were identified (miRBase v21) in the human genome and many of them are implicated in cardiovascular development, function, and disease [1]. Among all identified cardiovascular miRNAs, a heart-enriched miRNA called miR-208a has earned much attention not only as a critical player for the heart health and disease, but also as a potential biomarker and therapeutic target [2]. miR-208a is a member of a miRNA family that also includes miR-208b and is encoded by an intronic region of the* Myh6* gene that encodes *α*-myosin heavy chain, the predominant heavy-chain contractile protein in the adult heart [2]. Since miR-208a is a key modulator of many heart functions, alterations in its expression levels are frequently associated with pathological cardiac dysfunctions, such as hypertrophy, fibrosis, arrhythmias, contractile dysfunction, conduction abnormalities, fibrillation, and cardiac remodeling [2]. Indeed, an overexpression of miR-208a promotes deterioration in cardiac function, as indicated by decreased fractional shortening [3]. On the other hand, the therapeutic inhibition of miR-208a leads to a reduction in cardiac remodeling and improvement in survival and cardiac function during heart disease [4].

In this present issue, Tony and colleagues [5] reported very interesting findings regarding the potential of miR-208a silencing against doxorubicin-induced cardiotoxicity in mice. In brief, the authors administered 20 mg/kg of doxorubicin (DOX) as a single dose and after 7 days the mice hearts were harvested and analyzed. As a result, the authors showed a 4-fold increase in miR-208a expression and a pronounced downregulation of GATA4 in the control group. Meanwhile, the other group, pretreated with miR-208a antagomir, showed an attenuation of miR-208a expression and a restoration of GATA4 levels. On the other hand, pretreated mice showed an increase in the expression level of the antiapoptotic gene BCL-2 and a decreased apoptosis when compared with control group.

The miR-208a silencing described here is particularly interesting because DOX is the first-line drug in the treatment of many types of cancer. Despite its beneficial therapeutic effects, cardiomyopathy and heart failure are observed when DOX is chronically administered for several weeks [6]. Although several cardioprotective therapies have been proposed, cardiotoxicity remains a major concern of oncologists in cancer therapeutic practice [7–9].

Although there are some studies regarding the involvement of miRNAs in DOX-induced cardiotoxicity, to our knowledge, this was the first study to evaluate miR-208a as a therapeutic target in this issue. However, a contradictory point should be taken into consideration. In the study by Tony and colleagues [5], the authors observed an increase of 4-fold in expression level of miR-208a in mice hearts after the administration of a single dose of 20 mg/kg DOX while all other studies in this issue showed divergent results. Indeed, in a study conducted by Vacchi-Suzzi and colleagues [10], the expression level of miR-208a in mice hearts decreased during the DOX treatment (cumulative doses) similarly with its encoding gene* Myh6*. In parallel, miR-208b as well as* Myh7* were increased indicating a myosin switch which is associated with pathological cardiac remodeling. In the same way, Desai and colleagues [11] showed that after administration of 24 mg/kg of DOX the expression level of miR-208b was increased by 8.2-fold in mice hearts while no change was observed for miR-208a. The authors also did not observe significant effect on absolute heart weight or evidence of cardiac hypertrophy in histopathological evaluation. In a recent study, Nishimura and colleagues [12] showed that, after a single administration of a high DOX dose (30 mg/kg) in mice, the circulating level of miR-208a as well as cardiac troponins (cTnI and cTnT) did not change significantly while miR-1, miR-133a/b, and miR-206 were increased. In addition, no histopathological changes were observed in mice hearts. Finally, circulating miR-208a was undetected in plasma from breast cancer patients along the chemotherapy treatment with four cumulative doses of 60 mg/m^2^ DOX [13]. Taken together, these studies suggest that miR-208a is not upregulated by DOX at the acute stage of cardiotoxicity.

Another point to consider about the study by Tony and colleagues [5] is the lack of some methods which would have contributed to robustness of results such as the use of negative controls with scrambled antagomir. The authors also did not use lentiviral vectors making it difficult to assess the long-term duration of therapy. Since miRNAs are involved in a complex network of gene expression the potential effects of miR-208a silencing on epigenetic changes should have been assessed. These gaps make the data more limited providing only a partial interpretation.

It is also important to highlight that the authors adopted a short induction model of DOX-induced cardiotoxicity where the experiments were performed 7 days after the administration of a single high dose (20 mg/kg DOX). Thus, we should keep in mind the use of a chronic induction model with the administration of cumulative DOX doses for several weeks (e.g., 3 mg/kg/week), which represent a more clinical setting, might show different results.

Although the antimiR-208a therapy seems to be a promising tool to protect the heart against the DOX-induced cardiotoxicity, we must be very careful once this miRNA is a key modulator of gene expression in the heart and its silencing may lead to several cardiac abnormalities.
